# The role of web-based flipped learning in EFL learners’ critical thinking and learner engagement

**DOI:** 10.3389/fpsyg.2022.1008257

**Published:** 2022-10-20

**Authors:** Ya Pang

**Affiliations:** School of Foreign Languages, Hainan Normal University, Haikou, China

**Keywords:** social media, flipped learning, critical thinking, learner engagement, EFL learners

## Abstract

The flipped learning approach with the use of social media as an emerging technology has changed the quality of learning in English as a Foreign Language (EFL) educational contexts. This review probed the effect of the web-based flipped learning approach on learners’ engagement and critical thinking. The earlier studies revealed the significance of social media in developing learner engagement and critical thinking. Studies indicated that the provision of opportunities for more cooperative and collaborative learning activities, and high-quality interaction through the use of social media can be influential in developing learners’ engagement. Moreover, social media platforms can provide a context for feedback, and various types of challenging tasks that can improve EFL learners’ critical thinking. However, this review implicated that social media in flipped learning approach may be beneficial for instructors, learners, teacher educators, curriculum designers, educational policy-makers, and advisors to be aware of this valuable learner-centered approach.

## Introduction

In recent years, educators have used the technological developments in education to create a more effective learning environment in which learning does not seem to be limited to the classroom environment and teachers may not be obliged to spend most of the class time delivering lectures and instead, they can have tutorial roles while students can also take different roles and be more actively involved in the learning process (Tan et al., 2017). One of the instructional approaches that follows this technology-related learning approach is flipped learning because it utilizes technological tools, including recorded lessons and videos, to create more engaging experiences for learners. The concept of flipped learning is that the teacher-student roles are changed in a way that the amount of direct instruction presented by the teacher during class time is minimized while the cooperative and collaborative contribution of students to the teaching process is maximized in class (Bergmann and Sams, 2012). The flipped learning is an educational approach that inverts the operation of the conventional classroom by presenting the educational substances typically online, involving the students in cooperative group learning or potentially basic critical thinking exercises completed under the educator’s direction amid class.

Researchers have further revealed that with an effective design of learning activities, teachers can instruct students to comprehend, analyze, and even solve problems through discussion with classmates and teachers in flipped learning (Lin et al., 2021). From the perspective of social constructivism, interaction with peers implies better opportunities for sharing and constructing knowledge (Al-Qaysi et al., 2021). This implies that guiding students to interact with peers with proper support will enable them to perceive things from diverse perspectives and make reflections, which is beneficial for them to develop higher-order thinking capabilities, including problem-solving and critical thinking (Saputri et al., 2019). Students’ interactions with course content in all flipped learning environments can scale the top of Bloom’s cognitive taxonomy, breaking away from mere memorization and application to the more demanding steps of analysis, evaluation, and creation, the cornerstones of critical thinking (Jensen, 2019). Ferrett (1997) contends that critical thinking is the ability to think reasonably, reflectively, responsibly, and skillfully of and about whatever is around. It differs from the shallow naïve thinking in which the person does not deepen into the reasons for the events around. Critical thinking, according to Benesch (1993), can be employed and taught to second language learners of English as a crucial, preliminary strategy that not only heightens the concept of democracy among and within learners, it can help enhance learners’ awareness in learning language skills. Teachers ought to be aware of the capabilities in order to match technology with their instructional goals and they should know how to increase learners’ critical thinking, which in turn enhances learning outcomes (Jensen, 2019).

Another major issue for educators is related to how students can become more engaged before, during, and after the flipped class period. Different factors affect students’ engagement in the learning process, including teacher support, quality of instruction, peer connections, and classroom structure and management. By taking advantage of integrated technology by using it as a tool, the flipped classroom presents information prior to the classroom and, as a result, makes students more engaged with the course contents before attending the class. This review considers the studies on the effect of the web-based flipped learning approach on learners’ critical thinking and engagement. This conceptual review tries to specify important perceptions about web-based flipped learning approach and give some suggestions for future research. The result of this conceptual review can improve, reconceptualize, or even replaces current views of flipped learning approach. The findings and implications of the present review will provide relatively novel insights for teachers. Exploration in this field can help educators in many aspects of the classrooms to find new approaches to become more effective teachers and accordingly make a practical learning setting for increasing learners’ critical thinking and engagement in the educational context.

## Review of literature

### Flipped learning

Technology has become an integral part of educational environments today. As Lindeiner-Stráský, Lindeiner-Stráský et al. (2020) have suggested, the development of technology has significantly changed the ways instructors teach, and students learn. They mentioned that the teachers are willing to integrate technology into their approaches. This willingness causes a re-evaluation of teachers’ instructional styles. Indeed, they incorporate technology into their teaching in order to achieve their pedagogical goals better. Amongst the numerous ways of technological-based pedagogy practices in English language learning is the flipped learning approach (Yulian, 2021). According to Guo (2019), the flipped learning approach is a pedagogical approach that encourages students’ active participation, promotes support from teachers, and peers to handle homework, and allows more free time in class. Zainuddin (2017) mentioned that in a flipped classroom, learners acquire the knowledge before the class, and the class time is dedicated to practicing the knowledge through active learning and problem-based learning without spending time on presenting the content. Hung (2018) defined pre-class self-learning as the bringing of linguistic knowledge to learners’ private space and time with the help of videos and related exercises. He also described the in-class activities as pair or group activities during class time. Therefore, the shift of material consignment to the outside of the class and using the class time for higher-level activities like applying and examining the earlier learned materials are the primary components of flipped learning approach (Yilmaz and Baydas, 2017). A flipped classroom enhanced by Bloom’s revised Taxonomy and Cognitive Apprenticeship, gives EFL students more exposure, time, and opportunity to learn both in and outside the L2 classroom. It shifts teacher-driven instruction to student-centered learning through active learning strategies.

The theoretical foundations including constructivism (Aljohani, 2017) and cognitive load theory (Li, 2022) can justify the role of flipped learning approach by the instructors. The constructivist philosophical view of constructivism is knowledge created through communications with one another involving the society and setting (Rob and Rob, 2018). Based on constructivism theory, learning occurs when a student works either with a more skilled adult or peer to solve problems that are just beyond her/his actual abilities (Jantakoon and Piriyasurawong, 2018). Core principles of constructivism include the following: (1) learning is self-centered and self-directed; (2) learning is an active rather than passive endeavor; and (3) the instructor’s role is to foster critical reflection and facilitate the application and deeper understanding of new concepts (Aljohani, 2017). Student-centered instructional approaches, like flipped learning approach, are grounded in the constructivist theory of learning in which the learner is an active constructor of knowledge based on previous experience, perceptions and knowledge (Lewis et al., 2018). The constructivist approach in flipped learning should be an active practice, where learners must build their knowledge and make use of cooperative and collaborative learning, be given power in the learning procedure, be given chances to reflect and, lastly, gain meaningful learning experiences in order to enhance their learning based on this particular approach (Erbil, 2020). Flipped learning can contribute to the decrease of the cognitive load levels of learners. (e.g., de Leng and Pawelka, 2021; Li, 2022). Cognitive load means the resources used by an individual’s working memory at a certain time (Kirschner et al., 2018). Based on the cognitive load theory, efficient instruction should have a small extraneous load and an optimized germane load (Wang et al., 2020). Kirschner et al. (2018) stated that efficient instruction increases learning outcomes and decreases cognitive load. The flipped classroom approaches provide additional opportunities to manage cognitive load by allowing the learners to have knowledge about the subject before the lesson which improves learning. Some of these are implicit in the flipped classroom approach; however, others will require educators to make certain choices when designing learning activities.

The use of social media in flipped classrooms has drawn the attention of many investigators. Pathiraja and Little (2015), generally defined social media as a “set of interactive technology tools designed to encourage social networking and dialogic communication in virtual communities and networks” (p. 585). They mentioned that social media platforms include online forums, networking sites, online professional networks, content posting sites, and research forums. Bryer and Zavattaro (2011) also described social media as “technologies that facilitate social interaction, make possible collaboration, and enable deliberation across stakeholders” (p. 4). Veletsianos and Navarrete (2012) indicated that social media platforms can bridge the gap between technology and pedagogy in flipped classrooms. This review will discuss the role of social media platforms in EFL learners’ critical thinking and engagement in flipped educational contexts.

Vygotsky’s theory of mediation in digital learning is a theoretical construct of this review. Based on this theory, technology can be related to psychological and cognitive states. According to Zidoun et al. (2019), education programs should consider the role and impact of technological developments on learning. The concept of technological mediation, inspired by Vygotsky’s (1986) theory of tool mediation, aims to gain insight in the ways in which technology actively co-shapes the relation between people and the world through various mediating effects. de Boer et al. (2018) explain that this understanding of technological mediation emphasizes “the primacy of the relatedness between emotional states of people, technologies, and the world” (p. 300).

### The concept of critical thinking

Critical thinking is considered a fundamental component of educational activities (Thorndahl and Stentoft, 2020; Oktaviah et al., 2021). Many investigators have provided numerous definitions for Critical thinking. According to McPeck (1981), critical thinking is defined as “the propensity and skill to engage in an activity with reflective skepticism” (p. 8). Sternberg (1986) also considered critical thinking as a cognitive psychological component and defined it as “the mental processes, strategies, and representations people use to solve problems, make decisions, and learn new concepts” (p. 3). Gavrysh and Dotsenko (2021) declared that critical thinking is described as an individual’s ability to self-assess the surrounding, reality, information, knowledge, opinions and statements of others, and the ability to find effective solutions considering existing stereotypes and criteria. Yulian (2021) also regarded critical thinking skills as the main cognitive process dimension in Bloom’s taxonomy. According to this taxonomy, critical thinking includes remembering, understanding, applying, analyzing, evaluating, and creating Shubina and Kulakli (2020) asserted that critical thinking is viewed as the most common way of assessing thoughts, evaluating contentions, managing issues, making decisions, collecting and appraising different data, and concluding about particular principles to give the best solution. Tong et al. (2020) mentioned that critical thinkers reflect, relate and appraise all features of circumstances or problematic issues. They maintained that this level of thinking incorporates abilities like concentrating on components of a problem or an adverse situation, gathering and coordinating data about the problem, and recalling the understood information.

Bağ and Gürsoy (2021) stated that critical thinking skill is significantly correlated with foreign language learning achievement in which the learners are directed to determine their own motives to learn the language, set their goals consistent with their educational requirements, control their learning practice competently, and to employ proper skills of the era during the whole process. They also asserted that active cooperation, which is a vital issue in today’s world, needs competent thinkers, who can interact with others in a common language to critically analyze the messages, make reasoning, and inferences and create the meaning to express their own opinions. Itmeizeh and Hassan (2020) stated some of the features of critical thinkers, include “purposeful, self-regulatory, self-rectifying, habitually inquisitive, well-informed, trustful of reason, open-minded, flexible, fair-minded in evaluation, honest in facing personal biases, prudent in making judgments, willing to reconsider, clear about issues, orderly in complex matters, diligent in seeking relevant information, reasonable in the selection of criteria, focused in inquiry, and persistent in seeking precise results” (p. 2). Etemadfar et al. (2020) emphasized that good critical thinking is not an innate or natural ability for most L2 students, but it can be taught through effective pedagogical methods.

### The role of web-based flipped learning in developing learners’ critical thinking skills

The introduction of the flipped teaching method and digital technologies has the potential to encourage and promote active learning, learner-centeredness, and critical thinking skills of language learners in EFL classrooms (Pasaribu and Iswandari, 2019). Flipped learning with the help of technologies attracts students to their learning procedure through supportive and problem-based educational tasks to develop their basic reasoning and critical thinking skills (Prahani et al., 2020; Lin et al., 2021). van Vliet et al. (2015) stated that learners, involved in flipped educational contexts, tend to use critical thinking strategies, such as planning, monitoring, and evaluation strategies. Using Bloom’s taxonomy, Eppard and Rochdi (2017) justified the significant effect of flipped learning approach on learners’ critical thinking. They argued that, in flipped learning classrooms, the independent transmission of information by the learners outside the classroom and assimilation of information entails critical thinking, analysis, synthesis and reasoning. Likewise, Ebadi and Rahimi (2018) examined the effect of flipped and traditional educational contexts on Iranian EFL learners’ critical thinking. They used WebQuest, video clips and e-learning materials. Moreover, California Critical Thinking Skills Test Form B was used in order to evaluate learners’ critical thinking skills. Their study indicated that flipped classrooms, compared to non-flipped ones, foster learners’ critical thinking skills. Munir et al. (2018), in another study on the operation of a technology-based flipped classroom with the employment of cooperative learning, indicated that flipped classroom improves learners’ critical analysis skills, problem-solving skills, and communication skills. Cheng et al. (2019) also mentioned that flipped classrooms develop a student-friendly educational context that encourages learners to engage in a classroom making teachers assign and educational time for interaction by engaging learners in numerous interactive learning tasks, such as discussions, problem-solving, critical thinking, and hands-on activities. Zou and Xie (2019) investigated the impact of two technology-based flipped learning approaches, including just-in-time teaching and peer instruction in flipped classrooms and conventional flipped classrooms on EFL students’ critical thinking. Their study demonstrated that the technology-based just-in-time teaching and peer instruction flipped approaches are significant in developing learners’ inclination towards critical thinking. Kawinkoonlasate (2019) stated that the technology-based flipped learning approach is a reliable way for teachers to assign classroom time to foster critical thinking, self-directed learning, communication skills, and cooperation among the learners. He mentioned that technology-based flipped classrooms can obviate the academic weaknesses of some learners and ensure the development of their thinking skills. Afzali and Izadpanah (2021) also found a significant difference between technology-based flipped learning and traditional classrooms in developing learners’ critical thinking and performance. They argued that flipped instructional approach offers more communication, involvement, contribution, feedback, and various types of tasks inside the classroom context, which can foster learners’ critical thinking through integrating technology in the classroom by focusing on student-centered education.

Critical thinking strategies can enhance flipped learning. Kurnianto et al. (2019) underscored the role of motivation as a mediating variable in the relationship between critical thinking skills and learning in flipped classrooms. They mentioned that the increased motivation can significantly affect critical thinking and learning outcome in flipped classrooms. Self-regulation is also another component that develops learners’ critical thinking and learning in flipped classrooms. For instance, guiding learners to set their learning goals, and supporting them in monitoring their learning status in five stages, namely, goal setting, flipped learning (including pre-class video-based instruction and in-class discussion), task sharing, self-evaluation, and self-regulation feedback are influential in developing critical thinking and learning achievement (Chang et al., 2022).

Hamid et al. (2015) emphasized the importance of using social media as a platform in flipped learning, which makes increases interaction among learners. They stated that social media promotes learners’ engagement, self-monitoring, autonomy, and critical thinking. Van Den Beemt et al. (2020) focused on the importance of internet-based applications, like social media, in developing learners’ engagement and critical thinking. Puentes (2022) indicated that using social media develops environmental awareness, which generates a socially cooperative learning context and improves learners’ basic ecological critical thinking. Ariantini et al. (2021) found the significant roles of social media, such as YouTube, WhatsApp, Facebook, and Instagram, in learners’ language skills, vocabulary, grammar, pronunciation, spelling, motivation, as well as their creative, and critical thinking.

Andrini et al. (2019) indicate that the use of Information and Communication Technology (ICT) in the form of Moodle, simulation, distance learning, and utilizing social media (e.g., WhatsApp, telegram, line, etc.) in flipped classrooms, increases particular features of learners’ critical thinking skill, including elementary clarification, basic support, inference, and advance clarification. Moreover, they mentioned that the integration of social media into the combination of the flipped classroom and project-based learning, as a learning approach, enhances the communication and teamwork, and encourages the learners to use critical thinking to solve their project. He and Darmawan (2018) also emphasized the role of WhatsApp in flipped classrooms in developing learners’ higher-order thinking skills. They mentioned that digital-based flipped learning makes learners well prepared, more independent, and more active, which leads them to be critical thinkers. Listiqowati et al. (2022) investigated the effect of the project-based flipped learning approach on learners’ critical thinking skills. They used Zoom meetings and WhatsApp as platforms used in flipped classrooms. Their study revealed that the project-based flipped learning approach with the use of social media is influential in the enhancement of critical thinking abilities. Having used Zoom meetings and Facebook, El-Glil and Mohamed (2021) emphasized the role of social media in developing adults’ EFL critical thinking.

Regarding the importance of Facebook and Instagram in educational contexts, Al Arif (2019) asserted that the production of captions, pictures, and videos to be uploaded to the students’ Instagram and Facebook accounts are argued to have triggered creative thinking, along with critical captioning and commenting. He mentioned that creative and critical thinking is one of the most important benefits of Instagram’s and Facebook for EFL students. He also stated that the uses of Instagram and Facebook in flipped classrooms urge the teachers to shift their teaching styles to be more active, flexible, effective, and student-centered in order to sharpen their critical thinking skills. Atwa et al. (2022) used Facebook as a platform for showing the videos and preparing learners in flipped learning classrooms to investigate the effect of digital-based flipped classrooms on EFL learners’ critical thinking and academic achievement in four different subjects, including Science, Math, IT, and English. They found that flipped learning approach increased all learners’ critical thinking skills, and Math achievement was evident in flipped learning classrooms. They argued that digital-based flipped classrooms help learners to comprehend deeply, retain knowledge and to use their higher critical thinking skills, rather than lower skills such as memorization and repetitions. Dewi (2022) used Instagram as a social media in flipped classroom in order to examine its effect on learners’ critical thinking and writing performance. His study revealed that Instagram can pave the way for critical thinking by providing learners opportunities for commenting and uploading photos and videos. Moreover, he found that writing skill can improve by using Instagram. He argued that teachers, by using Instagram, are not required to handle the whole learning process meanwhile, they can take responsibility as a facilitator and guidance. It means students must be given an opportunity to think critically and increase their creativity about the issues which trend online. Tencent QQ is also proven as one of the social media tool which can improve critical thinking. Luo et al. (2021) indicated that the interaction between student-teacher, teachers’ feedback, class hour, and communications on Tencent QQ are necessary to keep students on the right track of developing thinking skills. He maintained that students, with various opportunities of communicating with the teacher, would not feel powerless or frustrated when facing difficult tasks, thus ensuring the achievement of the learning objectives.

### The concept of learner engagement

Learner engagement is described as learners’ “psychological effort and investment toward learning, understanding, or mastering the skills, crafts, or knowledge that the coursework is intended to promote”. Christenson et al. (2012) defined learner engagement “as effortful learning through interaction with the teacher and the classroom learning opportunities” (p. 1). Jung and Lee (2018) also defined learner engagement as learners’ use of mental energy and effort to achieve the desired performance. Learner engagement has a positive and significant relationship with academic achievement (Lei et al., 2018; Miller et al., 2021). Learner engagement is regarded as a construct of positive psychology (Derakhshan, 2021; Greenier et al., 2021; Wang et al., 2021; Derakhshan et al., 2022). Philp and Duchesne (2016) conceived engagement as a multi-dimensional construct: behavior, cognitive, emotional, and social. They mentioned that behavioral engagement has been measured with items about attention, participation, concentration, and homework completion; emotional engagement is conceptualized as the presence of positive emotional reactions to teachers, peers, learning content, and classroom activities; cognitive engagement is defined in terms of using deep learning strategies, persistence, and self-regulated learning. A fourth dimension, social engagement, was proposed to stress the importance of social interactions in learning. Reeve and Tseng (2011) also presented agentic engagement as another component of learner engagement. They defined it as learners’ contributions to the flow of teaching instruction. In other words, students with higher agentic engagement are active in giving instructors their suggestions regarding various aspects of teaching in order to improve their own learning experiences. Engagement offers a more practical approach to involving students in their language learning, especially in today’s digital age, where too many distractions might interfere with learners’ efforts even if they are motivated (Mercer and Dörnyei, 2020).

### The role of web-based flipped learning in developing learners’ engagement

Earlier investigations have indicated that learner engagement is a fundamental component of enhancing the quality of education. Numerous investigations have proved the significant correlation between learner engagement and flipped learning approach (e.g., Afzali and Izadpanah, 2021; Li, 2021; Lee et al., 2022; Teo et al., 2022). In flipped learning, two modes of learning, pre-class and in-class, are based on a distinctly different approaches to knowledge and learning. In the pre-class portion of flipped learning, learners are required to autonomously engage in learning online lectures and materials in their own time and pace before engaging in the in-class learning activities. In the in-class learning portion of flipped learning, learners are required to actively participate in learner-centered activities designed to help them construct their own meaning through a deeper process of inquiry and investigation. Studies on FL have proposed ways to engage learners both in individualized pre-class learning and collaborative in-class activities (Ng, 2016). Diemer et al. (2013) have noted that pre-class engagement and in-class engagement have different learning requirements and outcomes. In addition to the potential differences in learner pre-class and in-class engagement, differences in engagement may occur at behavioral, cognitive, and affective levels. These levels are a multidimensional construct encompassing behavioral, cognitive, affective dimensions (Fredricks and McColskey, 2012). The study of Jamaludin and Osman (2014) revealed that learners’ behavioral, emotional, cognitive, and social engagement tend to be high in flipped classrooms, since they have more opportunities to work with peers, share and understand each other’s ideas through group activities in flipped educational contexts. They showed that the flipped classroom allowed learners to regularly alter their ideas of English learning and accept the significance of improving their English communicative skills through collaborating with peers. Evseeva and Solozhenko (2015) also found that flipped classrooms foster learners’ engagements through various technological tools. They mentioned that in flipped learning approach, learners have already been familiar with the subject, and the main notions, and have had some preparation for the subject matter, which make them more engaged in classrooms. Subramaniam and Muniandy (2019) compared learners’ engagement in flipped and didactic educational contexts. Learners in the flipped classrooms were asked to watch a micro-lecture before coming to class and were involved in engaging activities in the classroom. Moreover, they used a Likert scale questionnaire, which consisted of four engagement constructs, namely behavioral, agentic, cognitive and emotional engagement constructs. They found out that learners in flipped classrooms are more engaged in terms of these constructs. Salimi and Karimabadi (2020) also indicated that learners’ level of academic engagement in flipped classrooms tends to be more than of learners in traditional classrooms. They argued that learners’ academic engagement as a positive emotional state is related to the innovation and distinctiveness of the flipped learning approach. They asserted that learners are uninterested in repetitive and traditional instructional aproaches, and the features of flipped classrooms, such as active learning, learner-centeredness, collaboration, and communication with peers and teachers, were all the notions that increase learner engagement. Fisher et al. (2021) also found that flipped learning positively affects learners’ engagement, performance, and satisfaction. They mentioned that involvement stirred by flipped classrooms is integrally satisfying to learners. They also mentioned that intensive and strategic engagement with electronic materials before class is the feature of learners who outperform in flipped classrooms. Lee et al. (2022) investigated the role of belief and academic capability in increasing learner engagement in the EFL context. They found that learners’ epistemological beliefs influenced neither pre-class nor in-class engagement, but that academic capability affected both pre-class and in-class engagement. Doo (2021), in his study, showed that flipped learning approach provides learners with flexible learning in their study, time to create individual learning, and to engage more in classrooms. However, the factors affecting learner engagement in pre-class and in-class flipped learning are not widely studied. Nevertheless, more research is needed on the relationships between each dimension of engagement and different types of flipped learning outcomes, as well as on the path by which each engagement predicts flipped learning outcomes. Moreover, more studies are required to investigate learner engagement after flipped classrooms.

Flipped learning approach enables learners to be more engaged in the educational context to achieve all levels of Bloom’s taxonomy (Gilboy et al., 2015). In flipped classrooms, student-centered goals are set and active learning strategies such as pair-and-share activities are employed in flipped classrooms. Moreover, student presentations and discussions, and individual or paired quizzes empower students to reach higher levels of Bloom’s taxonomy (DeLozier and Rhodes, 2017). Creating ideas as the highest tier of thinking engages students to show their professionalized skills (Jaenudin et al., 2020). Applied to social media, Bloom’s Taxonomy can be used to design exercises that facilitate specific objectives. For example, the act of searching for and retweeting course-related content engages students with course material outside of class on a platform that is already of interest. This bridges formal-informal learning and may also increase student engagement by providing an innovative assignment. Furthermore, studies show that personalizing the online learning experience is crucial to improve adult learner engagement and reducing their attrition rates.

The use of social media in flipped learning approach plays an important role in learners’ engagement. For instance, Schindler et al. (2017) indicated that Facebook and Twitter affect learners’ behavioral, cognitive, and emotional engagement. They argued that Facebook and Twitter are practical tools for increasing specific behavioral and emotional engagement indicators, such as interactions with others and a sense of belonging within a learning community. They also mentioned that the mandatory use of Twitter, increasing faculty involvement in Twitter, and integrating Twitter into assignments may help to increase learner engagement. In light of the pedagogical potentials of social technologies such as Telegram in designing a variety of collaborative and cooperative tasks Amiryousefi (2019) set out to examine the effects of two types of flipped learning conditions with the use of Telegram on EFL students’ speaking and listening abilities and their participation and engagement with the materials and activities. His study revealed that flipped learning can help EFL learners improve their L2 speaking and listening and be more engaged with materials and activities outside of class. He argued that social technologies such as Telegram can create a less threatening learning environment compared with conventional settings, provide opportunities for more cooperative and collaborative learning activities, stretch the limits of input and output, and hence increase opportunities for high-quality interaction. Through Telegram, as the online platform of the flipped learning conditions in his study, the learners collaboratively elaborated on the learning materials during the pre-class phase of flipped learning and were hence engaged in collective scaffolding. He maintained that learners, through collaborative and scaffolding learning, can achieve beyond what they can do individually with the help of their peers and/or teachers and hence, according to Vygotsky’s ZPD theory, can learn beyond their abilities. Regarding the importance of social media on learner engagement Liu and Moeller (2019) highlighted the significance of WeChat as a social media in flipped learning classrooms. They mentioned that WeChat provides asynchronous, semi-synchronous, and synchronous online interaction, and its messaging function allows for interaction in different modalities (e.g., text, audio, and video messages). They argued that its user-friendly grouping function enables language instructors to group learners in the way that is most appropriate for achieving the intended learning objectives. This flexible grouping approach promotes engagement in online interpersonal communication either in pairs or in groups at any time, any place. Yu et al. (2022) also proved the effectiveness of WeChat on developing learners’ behavioral, social, cognitive and emotional engagements compared with traditional classrooms. Some studies also proved the effectiveness of Tencent QQ, as a social media, on the enhancement of learner engagement. Luo et al. (2021) found the role of an online-based environment in promoting learners’ sense of belonging and engagement. They used Tencent QQ as a professional online education platform in China. Using Tencent, learners could communicate with peers and instructors in the group, and even add instructors as friends to realize one-to-one online learning guidance. Their study revealed that the harmonious relationships by Tencent improved learners’ sense of belonging and engagement in online learning. They argued that online learning environment through Tencent can provide an opportunity for learners to have learner- teacher and peer relationship, which in turn, increase learners’ affective engagement. Teng and Wang (2021) also verified the importance of Tencent QQ and Wechat as the most convenient social networking system since it can enable learners to communicate academically or non-academically, and it promotes learner engagement. Piki (2020) considered the importance of Messenger, Viber, and Facebook in developing learners’ language learning and nurturing learner engagement. He also asserted that the interaction on social media contributed to re-establish learner engagement which was challenged during the unforeseen circumstances caused due to the COVID-19 pandemic. Zen et al. (2019), in another study, found a significant relationship between flipped classroom method using WhatsApp and learner engagement in activities. In line with Zen et al. (2019), Badshah et al. (2021) revealed the role of WhatsApp as asocial media in learners’ engagement by making a productive relationship among the parents, teachers and principal in underdeveloped countries’ schools. They showed that improvements in the parental engagement and teachers’ engagement result in a productive learners’ engagement. They argued that interaction with parents and teachers through WhatsApp has remarkably enhanced the parents’ and teachers’ involvement in children’ education, resulting in productive learners’ engagement inside and outside the classroom. However, Reflianto et al. (2021) investigated the influence of online-based flipped classroom learning between using Microsoft Team and WhatsApp, and learner engagement on reading comprehension skills. They found out that Microsoft Team was better than WhatsApp in improving learner engagement and reading comprehension skills. They argued that great, online learning methods with complete learning media features presented by teachers have proved to improve active learner behavior during online learning. Lee et al. (2022) investigated the effect of Zoom in flipped learning classrooms on learner engagement. They found that a synchronous online flipped learning application, such as Zoom, can encourage learners to involve in numerous cooperative activities. In general, studies showed that social media can predict learner engagement, and it can account for learners’ involvement in educational contexts.

## Conclusion, implication, and suggestions for further research

This conceptual review aimed to extend past research in a particular domain in a meaningful, conceptual way. The results of this conceptual review revitalized of existing theories and explored the novel conceptual insights. This review probed the role of flipped learning approach in learners’ critical thinking and engagement in academic contexts. Erlier studies have shown that web-based flipped classrooms have the capability to increase EFL learners’ critical thinking (e.g., Munir et al., 2018; Cheng et al., 2019; Pasaribu and Iswandari, 2019; Prahani et al., 2020; Lin et al., 2021). Previous investigations indicated that social media can draw the attention of learners by having supportive and problem-based educational tasks to foster higher-order thinking and critical thinking skill (Cheng et al., 2019). Moreover, it was also concluded that social media, such as YouTube, WhatsApp, Facebook, Instagram, Telegram, Tencent QQ, and Zoom provide the context for cooperative learning, which fosters learners’ critical thinking skill. Using these types of social media platforms can develop learners’ ecological awareness, which in turn ldeas to the development of socially cooperative learning, and critical thinking. This review also concluded that social media platforms enhance learners’ critical thinking by engaging learners in numerous interactive learning tasks and they increase learners’ inference and clarification. Social media platforms make learning more independent and active which increase higher-order thinking among learners. Earlier studies have also indicated that social media platforms, such as Facebook, Twitter Telegram, WeChat, Tencent QQ, Messenger, Viber, and Zoom can develop learner engagement by activating learners in numerous cooperative activities, raising parents and teachers’ attention to learners’ engagement (Badshah et al., 2021), enabling learners to communicate (Teng and Wang, 2021), improving teacher-learner relationship (Luo et al., 2021) and providing the features for making user-friendly groups (Liu and Moeller, 2019). This review also concluded that social media platforms can develop collaborative and scaffolding learning, which improve learner engagement.

This review comprises some pedagogical implications for instructors, learners, teacher educators, curriculum designers, educational policy-makers, and advisors. Based on related literature, instructors should have an awareness of flipped instructional approach in their classes to increase learners’ critical thinking and engagement. As one of the major findings of the studies was the outperformance of the learners in the flipped classrooms, the ground must be provided for expanding and appropriate use of flipped learning approach in our educational system at both institutes and schools. Moreover, web-based technologies should be used in classes in order to improve the quality of learning and teaching.

Teachers can simplify the problem of learners in flipped classes by using appropriate social media in order to help learners become more proficient in different context. Because class time is just for practicing and problem-solving in flipped classes. However, when EFL learners know why they learn a language, they are more cautious about the ways that facilitate this process. Language teachers can employ social media networks extensively to compensate for the shortage of time and to share a variety of contents including pictures, texts, audio, and videos, using these platforms to provide learners with authentic materials for different language skills. Furthermore, using the flipped classroom in language teaching can benefit language teachers in various ways, as teachers can monitor learners’ communication and the way they use target language outside the classroom in those networks to find out difficult and challenging aspects of their language use, and focus language teaching in classroom on these features.

Flipped classrooms increase student engagement. This can be done through providing learners with some opportunities to do lower-order learning activities at their own time, and to perform higher-level tasks through collaboration during class time. That way, instructors can guide the students through exercises and get better insight into who understands the materials and who is struggling. Students have more opportunities to ask questions and get personalized help as they work through the material using higher-order thinking skills. To keep the learners’ attention going, educators can add quizzes, puzzles and creating an appealing experience for students. They should devote time to active, collaborative learning activities that help learners evaluate, analyze, and synthesize materials. In flipped classrooms, they can use remote collaborative learning activities to make a little more thought and preparation than in-person ones, but they are equally rewarding. Moreover, they can use classroom debates, and they can appoint learners to represent two sides of a timely or controversial issue, and have them present arguments defending their position. In addition, they can employ breakout discussions, and they can employ the class in smaller breakout rooms, and have students discuss a question, issue, or problem. At the end of the session, they can have each group report on their conclusions. They can also use jigsaw activities by breaking the class up into groups of four or five students. They can have each individual in the group research a different issue or component of the broader subject. During class time, they can have them come together and share their findings. They are recommended to employ seminars in their flipped classrooms, and they can get students to take turns leading a class discussion on a topic they have researched. In addition, instructors need to develop digital expertise to provide immediate feedback, adequate guidance, and strong support throughout the flipped instruction and to build inter-connectivity between pre-class materials and in-class tasks, based on the flipped approach. Moreover, to reduce learners’ workload, sufficient time should be given to learners in the pre-class phase, whereas learning strategies and time-management training should be provided to maximize learners’ time use. Teachers can manage the time in classrooms regularly, and learner engagement to arouse motivation. They are required to decrease learners’ foreign language anxiety and disengagement, and they need to increase learners’ motivation irrespective of educational problems in language learning environments to enrich L2 learning experiences. They should talk to learners about their internal and external motivation in online contexts to be aware of learners’ personality traits which help them to engage enthusiastically in flipped learning contexts. They can make their class interactive and motivated by asking challenging questions throughout the class. Through asking and answering questions, learners can be more engaged during the course and learn information efficiently. Collaboration is another way for teachers to increase learner engagement. Learners tend to engage in classrooms when they cooperate on class projects.

Critical thinking can be explicitly taught in the English class, as an accelerator of the thinking ability as well as the speaking ability of the learners. The explicit instruction of critical thinking in English classes, according to the review, can develop higher order thinking in the process of language learning. It is the teachers’ responsibility to encourage learners to use their thinking ability and learn to express themselves critically and creatively. He believes that teachers need to be more flexible in their teaching and try to pay more attention to learners’ attitudes, interests, and abilities. Moreover, this review recommends language teachers include appropriate tasks, and activities in reading courses to promote critical thinking skills, which then can result in reading comprehension improvement. Teachers’ positive attitude toward critical thinking and providing learners with explicit explanations of the importance of critical thinking in education and every aspect of their life are the factors that can enhance learners’ critical thinking.

Learners can also improve their critical thinking skills by applying some practical ways. If learners think about the major objectives of the course, their critical thinking skills can develop. The type of activities used in assessing language learners determines the goals of learning. Learners should engage in doing those activities which promote critical thinking skills and require them to think, cooperate, ask questions from themselves and others, etc. learners should ask question in flipped classrooms since asking questions enhances your critical thinking in learning. One question may lead to another, and that will further help in clearing learners’ concepts. Moreover, critical thinking skills for learners in flipped classrooms can be developed through social experiences. If a learner gets opportunities to participate in discussions, both online and offline, S/he must go ahead with it. This will help learners come across different perspectives, and introduce one new information to analyze and develop better communication skills. Learners should also practice active learning through understanding and not just by reciting it innumerable times. It is a method of learning that is based on an experiential approach. Active learning can be achieved through group learning, case studies, demonstrations, visual learning, etc. It is easy to remember information through examples and stories as they reflect the practical implications. Real-life examples, anecdotes, analogies, and facts help develop critical thinking skills.

In order to increase teachers’ abilities in doing so, critical thinking issues and training on how to use different methods and techniques of teaching critical thinking should be an essential part of any teachers’ training program. On the other hand, training instructors in asking appropriate higher-level questions to promote critical thinking is of great importance. Teachers’ effective use of questions and involving EFL learners in class discussions over challenging and appealing topics could engage them in critical thinking processes. Moreover, a teaching and learning environment that considers different and sometimes competing views are crucial in promoting students’ critical thinking skills. Tracher educators can hold workshops in pre-service and in-service teacher courses to talk about the importance of learner engagement in language learning. It is also suggested that teacher educators should highlight interaction tools, like mobile applications, which promote engagement based on the subject matter. This review recommends that teacher educators should have a positive view toward teachers and learners, and they should provide well-organized and inspiring teaching methodologies, which can construct a positive context for language learning, and increase learners’ engagement in the classroom.

This review can attract curriculum designers’ attention to consider critical thinking, as an effective element, in their program. Improving learners thinking ability through appropriate techniques and methods of teaching should be taken into account in designing learning curricula since these skills are teachable and learnable. In an EFL setting, curriculum must be redesigned in which critical thinking skills and techniques be implemented to all courses possible. In other words, the focus should be on critical questioning, critical reading, critical and creative writing and critical listening in all curriculum areas. In addition, the goal of an ideal language curriculum should go toward developing the art of critical thinking.

Educational policy-makers should hire experienced teachers, as the instructive experience can be an important issue for increasing critical thinking and learner engagement among learners. They can ask teachers to do their best within varied educational contexts. They should also provide critical thinking, creativeness, and motivation into the education in classrooms, which enhances language learning. They can hold academic workshops to help teachers increase learner engagement and critical thinking. They can provide internet-based facilities and positive learning contexts for increasing positive behaviors among learners. The importance of learner engagement can motivate advisors to expand their horizons to identify learners’ sources of engagement, and to suggest some strategies to engage more in classrooms.

Given the considerable findings regarding the benefits of FL pedagogy and the growing interest in their factors, it is necessary to investigate how pre-class/in-class and behavioral/cognitive/affective engagement is influenced by learner factors like academic capabilities, learner style, personality traits, epistemological beliefs, positive psychological constructs, and negative emotional states, such as foreign language anxiety, apprehension, boredom, and burnout. More studies are required to investigate whether flipped classrooms can sustain learner engagement after flipped classrooms or not. Theoretical underpinnings like dynamic motivation systems theory (Mercer and Dörnyei, 2020) can be suggested for further research, in order to illuminate the way multiple internal and external factors affect EFL learners’ engagement the flipped classrooms. There are some concerns about the asseesment of flipped learning. Some studies have been done the effect of using e-portfolios on the assessment of EFL learners’ speaking proficiency (Yastibas and Cepik, 2015; Khodi et al., 2022). Moreover, it is recommended to have students conduct self-evaluations and to apply peer evaluations to foster changes in students’ attitudes toward learning in flipped learning contexts (Kim, 2018). Future studies can be done on the reliability and validity of e-portfolios, self-evaluations, and peer evaluations in flipped contexts. Finally, the comparison of different social networks on learners’ critical thinking and engagement in a foreign language can be studied in the future.

## Author contributions

The author confirms being the sole contributor of this work and has approved it for publication.

## Funding

This study was supported by the “Research on the Reform of Foreign Language Specialty Training Mode in Local Normal Universities under the Background of New Liberal Arts,” a new liberal arts research and reform practice project of the Ministry of Education in China, 2021 (grant no. 2021110080), and by Hainan Normal University – “Research on Project-based Teaching Based on Flipped Classroom – A Case Study of Comprehensive English (grant no. hsjg2021-23).”

## Conflict of interest

The author declares that the research was conducted in the absence of any commercial or financial relationships that could be construed as a potential conflict of interest.

## Publisher’s note

All claims expressed in this article are solely those of the authors and do not necessarily represent those of their affiliated organizations, or those of the publisher, the editors and the reviewers. Any product that may be evaluated in this article, or claim that may be made by its manufacturer, is not guaranteed or endorsed by the publisher.
